# The Addio Pizzo movement: exploring social change using agent-based modelling

**DOI:** 10.1007/s12117-016-9288-x

**Published:** 2016-09-10

**Authors:** Corinna Elsenbroich

**Affiliations:** University of Surrey, Guildford, UK

**Keywords:** Agent-based modelling, Extortion rackets, Social change

## Abstract

Extortion racketeering is a crime that blights the lives of everyone in societies where it takes hold. Whilst most European countries have some form of extortion racketeering, in most countries it is isolated to some ethnic communities. In Southern Italy and Sicily, extortion racketeering is still a feature of overall society. This paper attempts to look at the phenomenon from the angle of collectives, of resistance building through civic organisations such as *Addiopizzo*. For this investigation a computational model is presented to analyse the effect of team-reasoning on levels of resistance in systemic extortion rackets. An agent-based model is presented that implements the interaction of different kinds of decision-making of extortion victims with law enforcement deterrence. The results show that established extortion rackets are hard to undermine unless bottom-up civic engagement and law enforcement go hand in hand.

## Introduction

Extortion is the demand for money (or favours) using the threat of violence. An extortion racket is the continuous, regular and systematic extortion of several victims by a criminal or (more usually) a criminal organisation. Extortion racketeering is a global phenomenon. In a report on the extortion phenomenon in EU member states, a detailed country-by-country comparison is given of levels of extortion, what kinds of extortions are prevalent, who are the perpetrators and who are the victims (Transcrime (2008)). Almost all EU member states have some kind of extortion but in most it is at low levels and largely confined either to particular ethnic groups or to the criminal underworld. There are however some places where extortion is endemic. One of theses places is southern Italy and Sicily where the Mafia organisations *Cosa Nostra*, *’Ndrangheta*, *Camorra* and *Sacra Corona Unita* have an extortive stronghold on several industrial sectors, such as the building trade and waste management. They also have a stronghold on territories where entrepreneurs, e.g. shop, restaurant or bar owners, are systematically extorted for protection money, called the *pizzo.* In most Latin American countries, drug cartels and gangs extort rural areas and the urban poor by the collection of protection money as well as rich people through extortive kidnapping. In Japan the Yakuza is represented in all walks of life up to the highest echelons of society, executing extortive practices to gain influence. In Russia and former Soviet countries the Russian Mafia maintains a stronghold over many industries. Worldwide motorcycle gangs, such as the Hell’s Angels and Bandidos, run protection rackets. For a typology and international comparison of extortion rackets, see Anzola and Elsenbroich (2015).

This paper starts from a recent development in Sicily, spreading throughout other areas of Italy affected by extortion rackets: the advent and rise of *Addiopizzo. Addiopizzo* is a civic movement originating in 2004 with seven students and since then having developed into one of the most important bottom-up resistance groups against the Mafia. Their initial campaign was to plaster the city of Palermo with stickers stating: *“A society who pays the pizzo is a society without dignity”*. Since then *Addiopizzo* has recruited increasing numbers of entrepreneurs, from small shops and restaurants to large cooperations, with a current membership of 1000 entrepreneurs and 12,000 consumers taking an *anti-pizzo* stance.^1^ Members pledge to no longer pay the *pizzo* and have their finances thoroughly investigated to verify the pledge.

“To get this certification, firms have to undergo a fairly lengthy review process managed by a committee comprised of university professors, entrepreneurs, and members of other anti-racket organizations.” (Vaccaro 2012, p. 28).

Once they pass, entrepreneurs become members and get a sticker they can display on their shop to show their anti-Mafia stance and to inform consumers that they are a *pizzo*-free shop. *Addiopizzo* also offers advise and help to entrepreneurs, supports denunciation of mafia activity, including participating in anti-mafia trials and runs programs educating about the Mafia (e.g. in schools). Despite the continuing success of *Addiopizzo*, around 80 % of businesses still pay *pizzo*, constituting an important revenue stream for the Mafia.

The beginnings of the Mafia are unknown but Gambetta (2000) points to a first mention of the Mafia in 1838 as an already established social force (Hess 1986, p. 114). Since then the Mafia reigned with impunity until this century; only now the bastion of power is slowly crumbling due to societal rejection of the Mafia, changes in legislature and increased law enforcement.

In this article we focus on two specific civic changes and two law enforcement interventions supporting the demise of the Mafia. Whilst for most crimes, crime reduction can focus on the criminals, e.g. by increasing deterrence or situational prevention, in the case of extortion the focus needs to also be on the victims, due to the parasitic and semi-collusive nature of extortion. One traditional way to do this was to punish collusion with extorters, trying to deter victims from paying protection money. For example in Italy, before 1992 entrepreneurs paying extortion money were often fined for collusion. The policy of deterrence was rather unsuccessful, partly because people feared the Mafia more than the Italian state. Since1992 the state has shifted away from deterring entrepreneurs towards supporting them to denounce extortion and resist extortion payment, by offering protection, relocation and even financial compensation (La Spina (2008)).

We present an agent-based model to test two kinds of decision-making of entrepreneurs regarding the payment of protection money. We investigate how the decline of the Mafia might come about by analysing the effect of behaviour changes and law enforcement interventions on levels of resistance. The paper is structured as follows: Section 2 discusses relevant literature on extortion rackets. Section 3 introduces the method of agent-based modelling and how it has so far been applied to the modelling of extortion rackets. In Section 4 an agent-based model of extortion racketeering is presented that implements different decision functions for entrepreneurs to pay the *pizzo* or resist. In Section 5 the model results are presented. The paper concludes in Section 6.

## Extortion racketeering

Extortion is a widespread phenomenon and one of the defining features of Mafia Type Organisations. Extortion is one manifestation of a variety of predatory crimes (Best 1982, p.108). It defies a classical categorisation in criminology, into crimes against the person and crimes against property as the transaction is monetary (property) but the threat can be either property or person.

In a quali-quantitative analysis of extortion cases in Sicily, the types of intimidation found were intimidation against property (39 %), against people (33 %), against people and property (6 %) and the remaining 22 % had either no intimidation or intimidation was not detectable. Comparing warning to actual damage, in the case of people 77 % were warnings and 23 % resulted in actual damage, in the case of property warning accounts for only 37 with 63 % resulting in actual damage, most likely arson (68 %); cf. Militello et al. (2014).

Another unique aspect of extortion is the openness in which it is pursued. Although extortion is clandestine in the sense that only the extorter and its victim know about it, the victim is fully aware of its situation and able to make decisions regarding the extortion outcome. These decisions are to pay up, to refuse payment or to negotiate a reduction in price, although only about 12 % of cases analysed in Militello et al. (2014) had any form of negotiation.

Despite its ubiquity extortion is a difficult crime to research. Given that the perpetrators are notoriously difficult to come by, much of the early research focussed on victims with survey research such as CENSIS (2003), SOS Impresa (2007), Euripsped (2007). Whilst trying to measure the quantitative aspects of extortion racketeering in Italy, these surveys did not enhance the understanding of extortion rackets. Similarly, qualitative interviews with victims are insufficient to produce anything at the level of generality that would help to understand either the problem or the extent of extortion. The main problem of victimisation research in this area is that levels of underreporting will be very high due to factors such as fear of Mafia retribution, of prosecution by officials for collusion or of shame for colluding.

Accurate statistics are not available and there is good reason to believe that even where data are available, levels of underreporting are potentially high affecting the viability of the datasets. Alternative empirical analyses can be found in La Spina (2008) and Transcrime (2013). They use multiple data-sources, including judicial documents, police reports etc. The research focus is shifted from the quantity of extortion to the understanding of the dynamics of extortion. This focus can also be found in the game theoretic models discussed below.

Transcrime (2008) provides a European wide comparison of extortion racketeering practices. EU memberstates are evaluated according to levels of extortion, the nature of the perpetrators and their victims. Types of extortion are distinguished according to whether the extortion is casual or systemic, and whether it is parasitic, symbiotic or predatory. Whilst the categories of casual and systemic focus on the actions of the perpetrators, parasitic, symbiotic and predatory shift the focus on the relationship between perpetrator and victim. However, in general predatory extortion goes hand in hand with casual extortion and symbiotic and parasitic extortion with systemic extortion. The reason is simple, if the perpetrator’s interest is short term and casual the extortion demand can be the maximum amount, even if this might destroy the victim. If the perpetrator’s interest is long term, looking for a systemic racket, there is an interest to keep the victim healthy, leading to parasitic or even symbiotic extortion.

This distinction leads to three types of victim behaviours, acquiescent, complicit and resistant (La Spina (2008)). Acquiescents are those silently paying the *pizzo* without reaping benefit, complicits pay but also benefit from the relationship with the extorter, e.g. by the extorter eradicating the competition or protecting the complicit from other Mafia organisations. These two victim behaviours result in parasitic and symbiotic extortion respectively. Resistants are extortion victims who decide not to pay. Militello et al. (2014) found the frequency of these behaviours to be roughly 60 % acquiescent, 27 % resistant and 6 % complicit; 7 % of cases could not be classified. One interesting result from Transcrime (2008) is that in most European countries, parasitic and systemic extortion are largely perpetrated within ethnic minority communities, terrorist organisations and the criminal underworld. The biggest exception is Italy (mainly Southern Italy and Sicily) where extortion is systemic and practiced on the whole population by long standing Mafia organisations. One explanation has been a very high level of distrust, towards the authorities as well as fellow citizens. Gambetta (2000) describes how the history of invasion created a culture of distrust and how this is the main stumbling block in fighting the Mafia. Gambetta (1993) provides an analysis of how the Mafia becomes a guarantor in market exchanges, ensuring the quality of goods and limiting fraudulent interactions. The Mafia becomes a regulating authority, replacing personal trust. But it is not only this personal trust that is lacking in Italy but also “systemic trust”, the trust that relies on the existence of laws and law enforcement. When the state is too weak to protect its citizens the Mafia fills the power void. Paoli (2001) identifies the unpopularity of the Italian State since unification in 1861 as one of the major causes in the difficulty of penetrating the rule of the Mafia.

Paoli (2001) points to a change in the social estimation of the Mafia, resulting from the judicial campaigns against Mafia and corruption. Since the 1990s the Mafia lost public support, partly due to the violent murders of the anti-Mafia Judges Falcone and Borsellino. Since the new Millennium the Mafia has become under increasing pressure by law enforcement and the judiciary as well as from civic society through organisations such as *Addiopizzo* which are evidence of a growing anti-Mafia sentiment in society.

But it is not only a lack of personal or institutional trust that is seen as an explanation for the emergence or persistence of extortion rackets. Extortion rackets have also been approached from an economic perspective. On a macroeconomic level one might look at the role extortion racketeering plays in an economy, either by analysing how the Mafia regulates markets (see above) (Gambetta (1993)) or following an analysis of underground and over-ground markets and their interdependencies in Schelling (1967). Schelling distinguishes between Black Markets and racketeering, providing a typology of organised crime endeavours. He discusses policy influences, such as prohibition, and analyses organisational structures of organised crime. He suggests that extortion rackets are simply a means to an economic end and contributes to a conceptual embedding of extortion dynamics into simple market dynamics.

A further economic aspect of racketeering is to analyse how the economic situation influences the success of racketeering. In particular unemployment and poverty have been linked to Mafia success as they provide a pool of recruits which cannot find legal employment (Lotspeich (1995)). In general, underdeveloped economies have been linked to Mafia infiltration although Calderoni (2011) points out that the Mafia is far from restricted to the economically underdeveloped regions of the South of Italy. Sung (2004) compares the economic failure hypothesis to the weak state hypothesis. State failure and economic failure were shown to be essential features of states infested by organised crime. In particular corruption of the judiciary and a thriving underground economy promoted organised crime whilst unemployment levels alone did not have a significant influence.

The micro level transactions in an extortion racket can also be viewed through economic spectacles, i.e. employing economic theory for the analysis (cf. Gambetta (1988, 1994); Varese (1994, 2001); Smith and Varese (2001)). This means to employ game theory for the analysis of the interdependent decisions of various players. The main focus has been the interaction between one extorter and one victim, i.e. a two-player-game (Smith and Varese (2001)) but there is also some work on the interaction between police and extorters (Konrad and Skaperdas (1998)). The decision focus is very intuitive as the extortion situations read almost immediately as a game theoretic decision tree, in particular in the case of the extorter/entrepreneur interaction. The extorter demands protection money from an entrepreneur, the entrepreneur decides to pay or not to pay depending on its expectation of being punished for resistance. If the payment demand is smaller than the probability of punishment multiplied by the possible damage, the entrepreneur will pay the extorter. Usually protection demands are small and, whilst a lot of punishments are symbolic or rather minor, potentially punishments are very high (e.g. arson attacks, murder). This means the important variable to assess for the entrepreneur is how likely it is to get punished by an extorter.

This game theoretic conceptualisation is the theoretical starting point of our ABM.

## Agent-based modelling

Agent-based modelling (ABM hereafter) is a relatively new methodology in the social sciences. It has successfully been used in disparate fields such as criminology, geography, organisation research, economics and sociology (cf. Squazzoni (2010), Liu and Eck (2008), Heppenstall et al. (2012), Watts and Gilbert (2014), Janssen and Ostrom (2006), Elsenbroich and Gilbert (2014)).

An ABM is a computer program that creates a world of autonomous, heterogeneous software objects, called  agents, in which each agent interacts with other agents and with the environment (Gilbert (2008)). For an agent to be autonomous means that its decisions are not made by a central decision maker (i.e. a programmer) but that the agent makes decisions depending on its own situation, goals, abilities, etc. Agents can be heterogeneous in their attributes, their situations, their roles and their behaviours. ABM is a particularly useful methodology for understanding social influence and neighbourhood effects, the interactions and interdependencies between different dynamics and dynamic developments of a system.

In Criminology ABM has been used for theory testing (Groff (2007); Johnson and Groff (2014), Birks et al. (2014)) and theory development (Birks et al. (2012)). The focus has been on theories such as rational choice, routine activity theory and environmental/situational approaches. A particularly interesting development for criminology is the integration of ABM and GIS which allows more detailed investigation of spatial patterns and crime dispersion (Liu and Eck 2008, Chapter 11).

There are some ABM exploring dynamics related to extortion, such as van Baal (2004, 2008) investigating tax fraud in such a way that agents decide on paying or not paying tax by evaluating punishments of neighbouring agents. Stewart and Plotkin (2012, 2013) discuss extortion in a slightly different definition where extortion is the exploitation of winning strategies in a Prisoner’s Dilemma.

More directly related ABM are presented in Troitzsch (2015), which discusses a model of extortion racketeering embedded in society to explore distribution effects. It is a systems perspective model in which individual decision-making is not taken into account. Interactions between the agent-sets of shops, consumers, extorters and police are investigated over a variety of input parameters, such as punishment-severity, denunciation-propensity and prosecution propensity (see also Troitzsch (2016) in this volume). Sonzogni et al. (2011) presents a model of Camorra operating in a specific region of Italy. The model investigates the effect of punishment severity and risk on compliance and collusion effects in different socio-economic and demographic environments. Nardin et al. (2016a) presents a model investigating the consequences to extortion rackets of changes to social norms. The model represents entrepreneurs as influenced by normative communication from the state and other entrepreneurs, based on the cognitive normative agent architecture Emil-A (Andrighetto et al. (2010)). Elsenbroich and Badham (2016) provides an ABM inspired by the game theoretic analysis of extortion rackets discussed in Smith and Varese (2001). This model shows the importance of implementing social aspects of extortion by comparing a decision-mechanism based only on an entrepreneur’s memory with a decision mechanism that takes into account what is happening in the neighbourhood of an entrepreneur.

In the next section an ABM of extortion racketeering is presented that explores the effects of different kinds of entrepreneur decision-making that might contribute to the erosion of established extortion rackets. The model in this paper contributes to the literature of agent-based models of extortion racketeering by implementing team reasoning, a reasoning mode which takes group membership and group payoffs into account. Team reasoning is a collective extension to individualistic rational reasoning, modelled in Elsenbroich and Badham (2016) and an alternative to normative reasoning modelled in and Nardin et al. (2016b), locating the motivation for behaviour change not in the recognition of a social norm but in the membership of a group.

## The model

The model presented in this paper is an abstract ABM. The decision rules are extrapolated from theoretical models like game theory and the numerical inputs, such as the levels of *pizzo*, damages, income, cost etc. are implemented with some proportionality rather than being informed by real data. Hence this model is far from a model calibrated and validated against data, able to produce policy advise on fighting the Mafia. What it does though is to systematically explore the effects of small changes in the decision function on overall levels of acquiescence and resistance. The decision function itself is informed by the intuition that entrepreneurs weigh up the danger and severity of being punished against the payment of *pizzo*, and although the actual values for punishment and *pizzo* are rather arbitrary, the decision function mirrors reality in that the potential cost of being punished is much higher than the *pizzo*, operationalising the fear factor Mafia type organisations exude.

The purpose of the model is to assist the understanding of the influences of normative and collective considerations together with law enforcement deterrence on levels of acquiescence in extortion racket systems. The model is implemented in NetLogo 5.0.1 (Wilensky (1999)) and is an extension of a basic model assessing the influence of neighbourhood effects on levels of resistance (Elsenbroich and Badham (2016)). This basic model demonstrates that extortion rackets can only become embedded in society if potential victims are able to observe (or otherwise learn about) implemented punishments. Whilst this model examined the emergence and persistence of extortion rackets, the model in this paper tries to understand their decline. For this it looks at the efficacy and interplay of two bottom-up decision mechanisms and two top-down law enforcement interventions. The bottom-up mechanisms are linked to the influence that civic organisations, such as Addiopizzo, might have on entrepreneur decision-making.

1. Bottom-Up Mechanisms
  Social Norms: Arguably in Mafia ruled areas of Italy, paying the *pizzo* is a social norm. Observing other entrepreneurs resisting the *pizzo* changes this perceived norm, enabling behaviour change. Civic organisations such as *Addiopizzo* might help to emphasise normative considerations. In the model this is implemented as normative reasoning by dividing the punishment probability by the number of resistants an entrepreneur observes in its neighbourhood.^2^
  Collectives: *Addiopizzo* is a membership organisation and might produce the feeling of belonging to a group. Group membership can have a profound influence on a person’s decision-making, e.g. engendering trust in cooperation (Sugden (1993)). In the case of an extortion racket, the difference might be to change from an individual perception of the situation to a collective perception. In the model this is implemented as collective reasoning by multiplying the cost of paying the *pizzo* by the size of the group an agent feels part of.^3^
2. Top-down Mechanisms
  Increasing Cost: If the Mafia can reign with impunity, unmolested by law enforcement, punishing resistant extorters is easy. However, if law enforcement hinders Mafia operations, punishment becomes dangerous, making extorters often unable to punish. In the model this is operationalised as a high cost of punishment. The cost is deducted from a Mafioso’s wealth after each punishment; if a Mafioso’s wealth is lower than this cost, it is unable to punish.
  Mafia Under Surveillance: Rather than making it difficult for the Mafia to punish, individual Mafiosi are taken out by surveillance so that they can no longer punish. In the model this is implemented by allowing some extorters to extort but not to punish. This is a reinterpretation of the idea of Fakers from Smith and Varese (2001) which is implemented in Elsenbroich and Badham (2016).

The model consists of a 61 × 61 torus-world of patches. There are two kinds of agents in the model, extorters and entrepreneurs. The model is initialised with 1000 entrepreneurs, randomly distributed on the world and 10 extorters, located in a circle with radius 15. The non-random allocation of extorters was chosen to reduce random effects resulting from clustering of extorters. Extorters approach entrepreneurs with a request for money, the *pizzo*. Once an entrepreneur agrees to pay, it pays this sum on a monthly basis. Each month (30 ticks) entrepreneurs assess the situation anew to decide whether to pay or refuse.

The decision to pay or not is made by weighing up the likelihood of punishment and the resulting damage against the cost of the *pizzo*. Entrepreneurs estimate the probability of punishment by observing punishments in their neighbourhood. If an agent observes a punishment its subjective probability is increased to 1, being divided by 1.1 each step no punishment is observed. The severity of punishment is set at 1,000,000 monetary units; this is deliberately a high value expressing the fear of Mafia punishment rather than the actual cost of real world Mafia punishments, which are often intimidation rather than actual damage (Militello et al. (2014)). The *pizzo* is set at 100 units, which is a realistic estimate if at the lower end of the actual demand.

The model is used to understand the influence of the four kinds of reasoning on the levels of acquiescence and the number of punishments. Tables 1 and 2 show the parameters of the simulation, what kinds of values they represent and for Table 2 which range of values they take in the experimentation.

**Table 1:** Parameters in the model that are held constant throughout experimentation

Fixed parameters	Value	Meaning
profit	400	Money paid to entrepreneurs every 30 ticks
pizzo	100	Value of protection payment
damages	1,000,000	Cost of being punished by Mafia
num-extorters	10	Number of Mafiosi
num-entrepreneurs	1000	Number of entrepreneurs
punishment-prob	0.5	Entrepreneur’s initial perception of punishment
prob-reduction	0.1	Increment punishment probability decreases

**Table 2:** Parameters used as variables in the experiments

Experimental-variables	Range	Exp-values	Meaning
neighbourhood-radius	1–30	5 10 15 20 25 30	Range of other agents considered
extorter-radius	1–30	5 10 15 20 25 30	Range of extorter territory
normative	True/False	True/False	Entrepreneurs reasoning normatively
num-collectivists	1–1000	1000	Number of collectivist entrepreneurs
group-radius	1–30	5 10 15 20 25 30	Size of collective
num-fakers	0–10	0 3 5 7	Mafiosi that cannot punish
cost	1–10,000	1 500 10,000	Criminal justice pressure on Mafia

The three horizontal groupings represent experimental parameters from the basic model, top-down interventions and bottom up change

One tick in the simulation represents one day; payday occurring every 30 ticks. Both entrepreneurs and extorters get paid on payday and entrepreneurs make the decision whether to pay their extorter or resist. On payday entrepreneurs obtain 400 monetary units in profits, so the *pizzo* is one quarter of the profit. Note that in this model neither the income nor the cumulative wealth of entrepreneurs plays a role in the decision function. In this paper the only purpose of entrepreneur payment is to transfer money to the extorter on a regular basis, dependent on the number of entrepreneurs on the payroll of the extorter. Once the extorter runs out of money they can no longer punish.^4^ Given the relative irrelevance of entrepreneur profits in this set of experiments they are kept homogenously at 400 monetary units.

Every day an extorter can make an extortion demand to one entrepreneur. If the entrepreneur is not yet extorted it decides whether to pay or not; if it decides to pay a payment link is established between the extorter and the entrepreneur, if it decides to resist a resistant link is established. If it is already extorted, nothing happens. Each step an extorter can punish one random entrepreneur linked by a resistant link. An extorter only punishes if it has enough money (wealth ≥ cost of punishment). After punishment the extorter’s wealth is updated as is the wealth of the punished entrepreneur. Entrepreneurs who are punished just live with debt in the current model.^5^

In the normative extension, agents observe levels of resistance in their neighbourhood and as resistance increases their subjective probability of punishment decreases, interpreting resistance as a new social norm. This is different from implementations of normative reasoning which is purely imitation or rule focused (e.g. Epstein (2000)). It is, however, in line with models where agents imitate agents with maximal payoffs (e.g. Axelrod (1997)). In our model entrepreneurs always consider the cost and benefit of acquiescence and resistance, rather than simply following others. The reason for this is that the punishments by the Mafia are potentially devastating, taking away the lively-hood of an entrepreneur or causing physical harm, making simple imitation or rule following unrealistic.

The collective extension is based on a collective extension of game theory developed by Bacharach and Sugden called *team-reasoning* (Bacharach (1999), Gold and Sugden (2006) Sugden (2003)). The idea is that although individuals maximise utility, they sometimes opt for maximising a group’s utility rather than their own. In the model this means evaluating not only the individual *pizzo* payment but the group *pizzo*.

The baseline reasoning for the model is the individualist strategic mode adopted from the model discussed in Elsenbroich and Badham (2016). Each agent *i* assesses the punishment probability P_i_ from its neighbourhood, multiplies it by the potential damage caused by a punishment and compares this to the *pizzo* that will have to be paid (Eq. (1)).

## Individualist strategic

1 $$ \begin{array}{l}\mathrm{If}\;{\mathrm{P}}_{\mathrm{i}}\times \mathrm{D}\ge \mathrm{E}\;\mathrm{pay},\\ {}\kern4em \mathrm{refuse}\ \mathrm{otherwise}\end{array} $$

Normative reasoning amends the left hand side of the equation by dividing the punishment probability by the number of resistants observed in the neighbourhood (Eq. (2)). Both the punishment probability and the resistance levels are observed within a given neighbourhood radius.

## Individualist normative

2 $$ \begin{array}{l}\mathrm{If}\;{\mathrm{P}}_{\mathrm{i}}/\;{\mathrm{R}}_{\mathrm{i}} \times \mathrm{D}\ge \kern0.37em \mathrm{E}\;\mathrm{pay},\\ {}\kern6em \mathrm{refuse}\ \mathrm{otherwise}\end{array} $$

Collective reasoning amends the right hand side of the equation by multiplying the *pizzo* by the number of agents in the group (Eq. (3)). The cost is now not just the individual’s but the cost of paying the *pizzo* is calculated for the whole group. Note that although the equations for normative and collective reasoning look mathematically equivalent, they are different as G_i_ is a fixed value throughout the simulation whilst R_i_ varies depending on the levels of resistance.

## Collectivist strategic

3 $$ \begin{array}{l}\mathrm{If}\;{\mathrm{P}}_{\mathrm{i}}\times \mathrm{D}\ge {\mathrm{G}}_{\mathrm{i}}\times \mathrm{E}\;\mathrm{pay},\\ {}\kern6em \mathrm{refuse}\ \mathrm{otherwise}\end{array} $$

To combine normative and collective reasoning both sides of the equation are amended (Eq. (4)).

## Collectivist normative

4 $$ \begin{array}{l}\mathrm{If}\;{\mathrm{P}}_{\mathrm{i}}/{\mathrm{R}}_{\mathrm{i}}\times \mathrm{D}\ge \kern0.37em {\mathrm{G}}_{\mathrm{i}}\times \mathrm{E}\;\mathrm{pay},\\ {}\kern7em \mathrm{refuse}\ \mathrm{otherwise}\end{array} $$

Equations (1)–(4) are summarised in Table 3, the matrix style representation is inspired by work on context dependent decision-making in ABM (Elsenbroich and Verhagen (2015)). Each reasoning represented here is dependent on environmental factors; the neighbourhood radius determines the punishment probability P_i_ by representing the neighbourhood in which punishment is observed. For normative reasoning the neighbourhood radius also represents the area in which resistance is observed to extrapolate a social norm. For collective reasoning the group radius determines the size G_i_ of the group the entrepreneur *i* considers in the calculation. The average number of agents in the set for varying radii is mapped in Table 4.

**Table 3:** The matrix of decision functions, where P_i_ is the probability of punishment for entrepreneur i, D the potential damage caused, E the *pizzo*, R_i_ the number of resistants observed by i and G_i_ the size of the group agent *i* feels a member of

	Individualist	Collective
Strategic	P_i_ × D < E	P_i_ × D < G_i_ × E
Normative	P_i_ /R_i_ × D < E	P_i_ /R_i_ × D < G_i_ × E

**Table 4:** The average number of agents in a neighbourhood or group for the sampled radii

Radius	5	10	15	20	25	30
# of agents	20	75	180	325	515	755

Two things need to be noted in this implementation of team reasoning:

- the payoff of all group members is considered equally;
- the group sizes experimented on is large.

The reason for the uniform consideration of group member payoffs is that there are currently no criteria to make a distinction. Such criteria would either result from different agents being more or less deserving/important or from relationships between agents, e.g. relationships of trust, friendship, and neither are modelled here. The relatively large group size is a result of a sweep of the parameter space. Smaller group sizes do not show any change in behaviour of the model, hence they are not reported here. We recognise that the large group size might be controversial, in particular for theories of team-reasoning relying on intergroup trust bonds (cf. Sugden (2003)). Trust bonds diminish rapidly with increasing group size (cf. Soboroff (2012)). We do nonetheless think this implementation is adequate as we are investigating an institutionalised group resulting from *Addiopizzo* membership rather than informal allegiances.

Elsenbroich and Badham (2016) showed the importance of the ability to punish sufficiently for the Mafia to keep control over their extortion territory. Although they do not have to punish a lot, if resistance is not punished sufficiently, resistance gets out of hand and the extortion racket falls apart. Normative and collective reasoning have been implemented to explore changes to levels of acquiescence brought about by entrepreneurs interpreting their contexts differently. Two law enforcement mechanisms that undermine the Mafia’s ability to punish are implemented. The first one is to increase the cost of a punishment. As discussed above, the cost should not be read as a simple monetary cost. Punishing a resistant extorter means a Mafioso exposes itself to possible capture by the police. The cost of punishment should be interpreted as an overall pressure exerted by the law enforcement agencies. The second way the police could undermine punishment is to put some Mafiosi under surveillance so they can no longer punish. This is implemented by making a number of the extorters unable to punish.

## Results

The results focus on the levels of acquiescence to pay the pizzo over a range of reasoning and law enforcement contexts. The model was initialised with an extortion racket in place, i.e. those in the territory of an extorter were paying *pizzo*. In the experiments the model ran for 1000 steps, with five runs over each parameter combination.^6^ The analysis was done over the dataset recording the simulation output for each step of the simulation. The data was curtailed to contain steps 300-1000 to eliminate remaining run in effects.

**Fig. 1:**
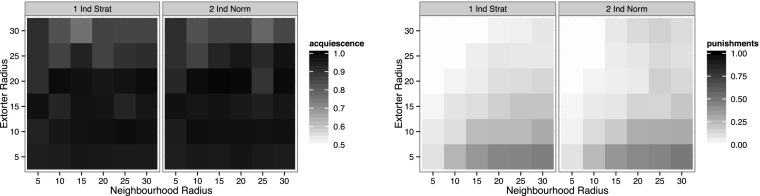
Acquiescence and average punishments per extorter per step for individualist strategic and normative reasoning

The baseline context is the individualist strategic context from Elsenbroich and Badham (2016). In this context acquiescence is consistently high, paired with low levels of punishment. The first amended context is an implementation of individualist normative reasoning (see Section 4 for a description). Figure 1 shows that individualist normative reasoning has very little impact on either acquiescence or levels of punishment.

This is a fairly surprising result as one would expect a rise in resistance for large neighbourhood radii. The reason behind it is that extorters keep such good control over their territory that levels of resistance never get to a critical point to influence the entrepreneurs normatively at a large enough scale.

**Fig. 2:**
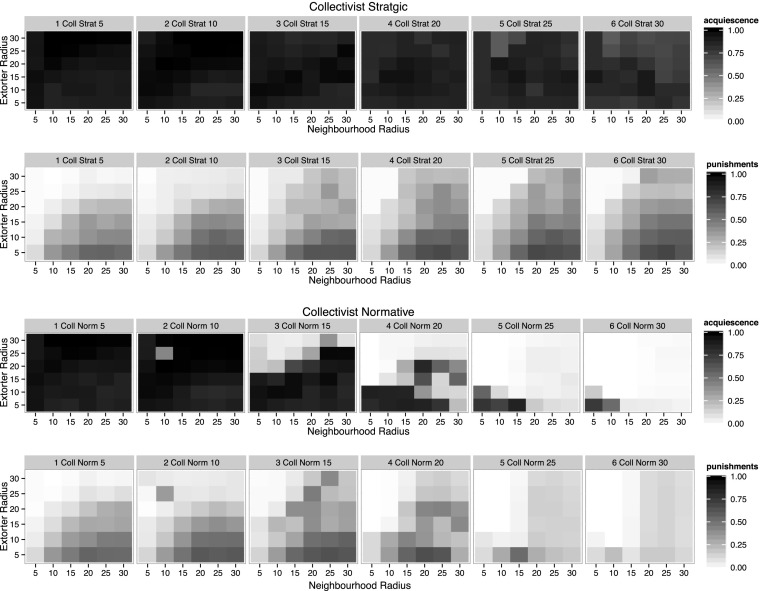
Acquiescence and average punishments per extorter per step. The *rows* show acquiescence and punishments for collectivist strategic and collectivist normative reasoning respectively. The columns show varying group radii between 5 and 30, increasing in increments of 5

Figure 2 shows levels of acquiescence and punishment for collective strategic and collective normative contexts over a variety of group radii, columns 1–3 showing strategic, column 4–6 showing normative reasoning. For collectivist strategic contexts a slight increase in resistance can be observed for high group radii (≥25) and high extorter radii (≥ 25) (see row one, columns 5 and 6). The reason behind this is that in larger territories the Mafia starts losing control. This is a result from the model only allowing the Mafia to punish one entrepreneur per time step; if in a large extortion territory several agents start to resist, one punishment per time step might not be enough to rein them all in again. Thus the punishment probability is lower for some agents in the extended territory which, combined with the high pizzo cost resulting from the large group, leads to increasing resistance. The effects, however, are small.

For collectivist normative contexts the impact for increasing group radii is significant. Figure 2 row three, column three shows that for a group radius of 15, acquiescence breaks down for large extorter radii, in particular for lower neighbourhood radii. This effect is strong, leading to almost complete resistance at group sizes of 30 (row three, column six), except for very small extorter and neighbourhood radii. In the strategic case the reason for very low levels of punishment in the combination of large extorter radii and small neighbourhood radii is that the population is acquiescent (see row two). In the normative case the pattern of punishments looks similar, but as here it is paired with high resistance, the cause of the low levels of punishment is different: Mafiosi have run out of resources to punish, rather than it not being necessary to punish (see row four, columns four, five and six). Whilst normative reasoning made very little difference in the individualist case, in the collectivist case it brings about a runaway effect of resistance, leading to the Mafia running out of money.

Given that the Mafia looses control of extortion rackets due to running out of resources, it is worth investigating the role of the cost of punishment in bringing the Mafia down. The collectivist normative case showed the possibility of an extortion racket being brought down by them running out of resources at a static cost level of 500. In the following experiment the cost is evaluated for values 1, 500 and 10,000; the group radius is kept constant at 15 (which was the first group radius which showed changes).

**Fig. 3:**
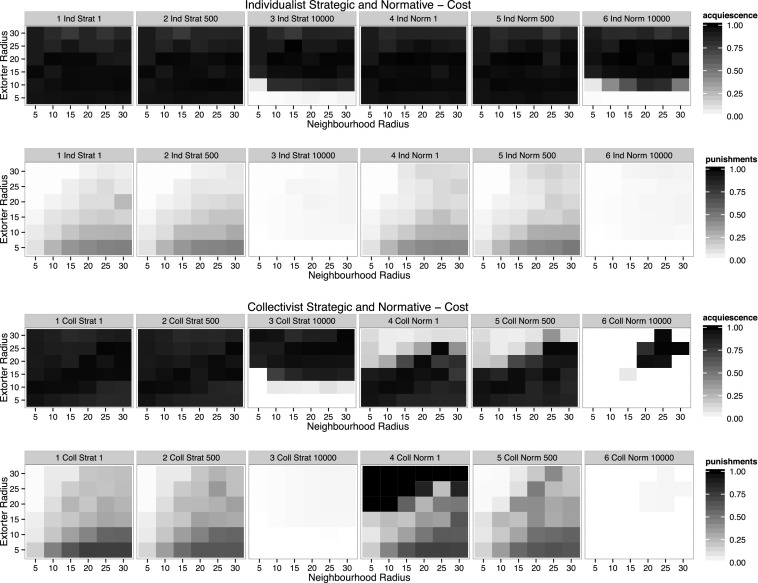
Acquiescence and average punishments per extorter per step. Row one shows levels of acquiescence, row two levels of punishment for individualist strategic and individualist normative reasoning. Row three shows levels of acquiescence, row two levels of punishment for collectivist strategic and collectivist normative reasoning. The columns differentiate levels of cost sat at 1, 500 and 10,000. Columns 1–3 represent strategic reasoning, columns 4–6 normative reasoning. The group radius is held constant at 15

Figure 3 shows that for individualist strategic and normative settings the increase in cost for punishment leads to almost no reduction in acquiescence except for very small extorter radii. If the extorter territory is too small an extorter does not have enough potential victims to regain the money needed for the next punishment. In higher extorter radii the effect of high acquiescence resulting from very low levels of punishment is emphasised. In the collectivist strategic case resistance can be achieved for an extorter radius of 10, even at high cost, but otherwise the same pattern of extremely low punishment and complete acquiescence can be observed.

In the collectivist normative case, a reduction of cost of punishment leads to higher levels of acquiescence. However, for large extorter radii acquiescence is very low, despite the high levels of punishment. For the case where punishment is restricted by a cost of 10,000, full resistance is reached and the Mafia can no longer punish.

As discussed in Section 4 the second law enforcement intervention to be explored is putting some Mafiosi under surveillance, i.e. allowing those extorters to extort but not to punish.

**Fig. 4:**
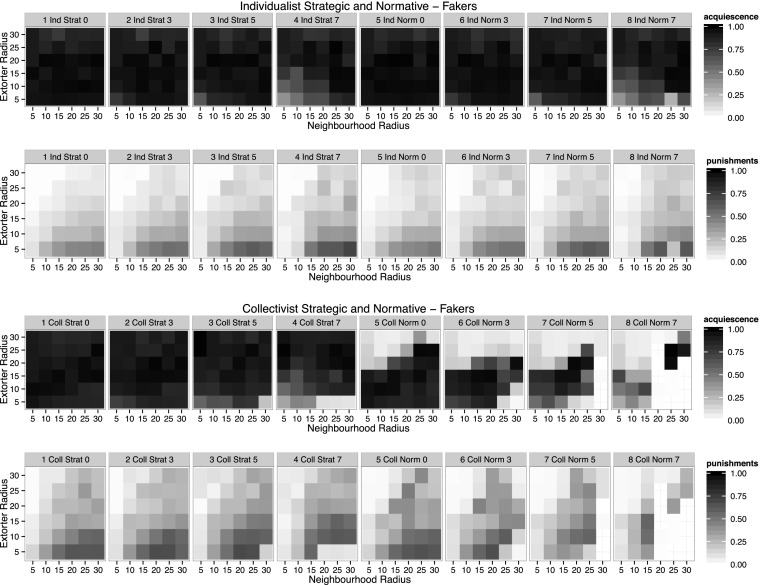
Acquiescence and average punishments per extorter per step. Row one shows levels of acquiescence, row two levels of punishment for individualist strategic and individualist normative reasoning. Row three shows levels of acquiescence, row four levels of punishment for collectivist strategic and collectivist normative reasoning. The columns differentiate 0, 3, 5, 7 Mafiosi under surveillance. Columns 1–3 represent strategic reasoning, columns 4–6 normative reasoning. The group radius is held constant at 15

Figure 4 shows similar patterns of levels of acquiescence, only reducing in the collective normative case. However some interesting facts need to be noted. One is the reduced acquiescence at low extorter radii. The lower the number of punishing Mafiosi, the more likely it becomes that there is a loss of control. Keeping control of one’s territory as a Mafioso under surveillance is to rely on your victims being deterred by other extorters. If the territories do not (sufficiently) overlap it is impossible for Mafiosi that cannot punish to live in the shadow of others (see also the analysis of Fakers in Elsenbroich and Badham (2016)).

For the collectivist normative case putting some Mafiosi under surveillance has very strong effects. Even taking three Mafiosi out leads to significant reduction of acquiescence, in particular for higher extorter radii where even punishing Mafiosi can loose control. For increasing neighbourhood radii the effect is surprising as one would expect acquiescence to increase with neighbourhood size but this only happens for very high radii.

## Conclusion & future work

This paper presented an agent-based model for the systematic exploration the undermining of established Mafia’s extortion racketeering. The underlying question is about civic (bottom-up) and law enforcement (top-down) ways of undermining extortion rackets. The model presented is an extension of an agent-based model investigating neighbourhood effects of deterrence on levels of acquiescence (Elsenbroich and Badham (2016)), finding that socially transmitted deterrence is essential to the flourishing of extortion rackets. In Elsenbroich and Badham (2016) entrepreneurs take information about punishments of neighbouring entrepreneurs into account when deciding to pay the pizzo or not. In the model discussed here entrepreneurs use information about punishments but also take resistance of neighbouring entrepreneurs and team members into account when making a decision. Whilst socially transmitted deterrence is essential for the flourishing of extortion rackets, this model shows the impact socially transmitted resistance can have on extortion rackets.

Socially transmitted resistance is implemented via two additional kinds of reasoning that might impact on levels of resistance in a population. The first reasoning is for entrepreneurs to reason normatively. Normative reasoning amends the utility function by reducing the punishment probability according to the number of agents in the neighbourhood that resist a racket. Collective reasoning amends the utility function by increasing the *pizzo* that is weighed up against the punishment to the *pizzo* of the whole group.

The results show that neither of the amendments on their own lead to significant changes, except for very high group radii. In general this poses a problem for collective reasoning aspects as often collective reasoning relies on trust and trust diminishes with increasing group size. In the case of extortion rackets the collective is not an informal groups but with organised resistance, in the form of e.g. *Addiopizzo,* meaning trust might be maintained with increasing group size. The more interesting result is how the combination of normative and collective reasoning brings about significant changes to resistance levels. These changes start occurring from medium group sizes (180 agents).

The reasoning amendments were implemented across two law enforcement interventions (in line with the original model). The first one, to increase the cost of punishment, has surprisingly little effect on extortion rackets, even though the cost constrains punishments. The computations show how few punishments might be sufficient to maintain an extortion racket. The second one is to take out a number of Mafiosi by surveillance, meaning they can still extort but no longer punish. The intervention shows results for individualist strategic and normative and collectivist strategic only for very low extorter radii (i.e. not very established extortion rackets). In established extortion rackets those that cannot punish can live in the shadow of Mafiosi still able to punish. In the collective normative case, hindering some Mafiosi from punishing has major effects. Taking out 30 % of Mafiosi is sufficient to reduce acquiescence significantly. The reason is that those Mafiosi still able to punish have insufficient ability to punish large waves of resistance.

There are, as always, limitations to this research. Models, by definition, are representations of reality and these representations can be at differing levels of abstraction. The model discussed in this article is an abstract model investigating a particular interdependency of neighbourhood effects, resistance and deterrence of Mafiosi. Models at this level of abstraction are often derided as “toy models”, bearing no relationship to the real world. Although abstract models are not calibrated against or validated by real world data, they are nonetheless useful as a tool to investigate dynamics and interdependencies that exist in the real world. Abstract models allow for the systematic exploration of a limited set of interdependencies and dynamics by limiting the parameter space. Whilst having limited empirical validation, abstract models reduce opacity. The model in this paper fixed a set of parameters, e.g. *pizzo*, punishment, to simplify the exploration of the relevant dynamics. This loss of realism resulting from the isolation of the reasoning mechanisms was a conscious decision to increase the transparency of the model.

In future work we are looking to use the findings of the possible importance of groups and team-reasoning and integrate them in data driven models of extortion racketeering, such as Troitzsch and Nardin et al (in this volume). ABM has so far focussed on individualistic approaches to social interaction and the model presented here is one of the first implementations of collective reasoning into an ABM (cf. Elsenbroich and Verhagen (2015)) and this model has shown that team reasoning is a potentially important aspect when analysing real world phenomena such as extortion rackets.

## Footnotes

1 Cf. http://www.addiopizzo.org/english.asp [accessed 11/05/2016]

2 The definition of normative reasoning used in this paper is rather specific and one reviewer pointed out that what is implemented is really just another version of instrumental reasoning. The existence of genuine normative reasoning is an unresolved debate. Much of normative reasoning in ABM has been implemented as a “socially informed rationality”, for example as norms establishing by following the agent’s strategy with the highest payoff (e.g. Axelrod (1986)). In this model I followed the idea of normativity being socially informed rationality, to not introduce another “innovation” into this ABM. Innovative approaches to normative reasoning in ABM can be found in Troitzsch (2016) and Nardin et. al (2016) in this volume who based the modelling on the EMIL architecture, the most genuinely normative implementation in ABM (Andrighetto et al. (2010)).

3 Note that all payoffs are equally important to the collectively reasoning entrepreneur. Although this might seem counterintuitive it follows the definition of team reasoning in Gold and Sugden (2006) and Sugden (2003).

4 This transaction is nonetheless implemented in the model using a regular payday for entrepreneurs so that the model can be used in future work to measure for example differences in equity between individualist and collectivist entrepreneurs.

5 The effects of individual entrepreneur’s wealth will be explored in future work to investigate different strategies of parasitic extortion.

6 Within parameter variation was found to be low in test runs, deeming five runs sufficient for robustness
